# Worms: Pernicious parasites or allies against allergies?

**DOI:** 10.1111/pim.12574

**Published:** 2018-08-29

**Authors:** Henry J. McSorley, Mathilde A. M. Chayé, Hermelijn H. Smits

**Affiliations:** ^1^ MRC Centre for Inflammation Research Queen's Medical Research Institute University of Edinburgh Edinburgh UK; ^2^ Department of Parasitology Leiden Immunology of Parasitic Infections Group Leiden University Medical Centre ZA Leiden The Netherlands

## Abstract

Type 2 immune responses are most commonly associated with allergy and helminth parasite infections. Since the discovery of Th1 and Th2 immune responses more than 30 years ago, models of both allergic disease and helminth infections have been useful in characterizing the development, effector mechanisms and pathological consequences of type 2 immune responses. The observation that some helminth infections negatively correlate with allergic and inflammatory disease led to a large field of research into parasite immunomodulation. However, it is worth noting that helminth parasites are not always benign infections, and that helminth immunomodulation can have stimulatory as well as suppressive effects on allergic responses. In this review, we will discuss how parasitic infections change host responses, the consequences for bystander immunity and how this interaction influences clinical symptoms of allergy.

## 1 KEY ELEMENTS IN TYPE 2 IMMUNE RESPONSES

Both allergic disease and helminth infection are associated with type 2 immune responses. Type 2 immune responses are generally required for control of parasite infections and have the advantage of causing reduced collateral damage compared with Th1 or Th17 immune responses. Conversely, in allergy, type 2 responses cause pathology which can be debilitating or even fatal. Models using mice deficient in key elements of type 2 immune pathways have shown the critical role of these responses in parasite killing/ejection, healing, metabolic changes and in allergic pathology.

Type 2 immunity is characterized by the development of antigen‐specific IgE immunoglobulins and Th2 cells producing IL‐4, IL‐5 and IL‐13. Upon recognition of antigen, Th2 cell cytokine production leads to the activation of eosinophils, while cross‐linking of IgE on primed mast cells leads to their degranulation. Together these effector immune cells are responsible for the clinical symptoms of allergic diseases such as atopic dermatitis, asthma and food allergy.

Interestingly, in both chronically helminth‐infected people and individuals who have experienced repeated clinical or environmental exposure to allergen,^1^ high antigen‐specific IgG4 levels can be found, as well as increased circulating levels of regulatory T cells (Treg) and regulatory B cells (Breg), producing IL‐10 and TGF‐β.^2^, ^3^ Thus, the type 2 adaptive immune response is capable of being tolerized, either through exogenous factors acting on adaptive immune cells, intrinsic exhaustion of those cells or changes in the innate immune system which is required for their activation.

Dendritic cells (DCs) are an innate immune population absolutely required for the development of optimal effector Th cell immune responses, including Th2 responses.^4^, ^5^ DCs are intimately associated with barrier sites such as the lungs and will take up antigens from within and beyond the epithelial barrier. Upon detection of a helminth infection or an environmental allergen,^6^ DCs become activated and migrate to the draining lymph nodes, presenting antigens to T cells and potentially inducing a Th2 immune response. Although the critical involvement of DCs in Th2 development is clear, the precise signals that lead to priming of a Th2‐inducing DC are incompletely characterized. In recent years, the importance of epithelial‐derived cytokines such as interleukin‐25 (IL‐25), IL‐33 and thymic stromal lymphopoietin (TSLP) in allergy and parasitic infection has become appreciated.^7^ These cytokines can act directly on DCs, skewing resultant responses to Th2, and also directly activate type 2 innate lymphoid cells (ILC2s), inducing a rapid innate type 2 response.

ILC2s are innate lymphocytes, lacking antigen‐specific receptors, which produce large amounts of type 2 cytokines IL‐5, IL‐13 and IL‐9, as well as proresolving factors amphiregulin^8^ and IL‐10.^9^ Activated ILC2s also express class II MHC, can present peptide antigen and can supply IL‐4R signals in type 2 response initiation.^10^

Thus, type 2 innate epithelial cell cytokines, DC and ILC2s are involved in the earliest responses to allergens and helminth parasites, in initiation of the Th2 immune response, and in amplification of allergic and antiparasite immunity. However, antigen specificity and the control of ongoing immune responses are critically dependent on adaptive immunity.

## 2 HELMINTH INFECTIONS, DAMAGE AND TYPE 2 IMMUNE RESPONSES

While type 2 immune responses have clear pathological roles in allergy, they are generally beneficial in helminth infections. Increased susceptibility to a range of intestinal and tissue‐dwelling parasites can be seen in mice lacking essential elements of the type 2 response pathway.^11^ Likewise, in human populations, single nucleotide polymorphisms (SNPs) in type 2 response elements such as IL‐13 and STAT‐6, and immunoregulatory elements IL‐10 and TGF‐β correlate with both decreased susceptibility to allergy and increased susceptibility to parasitic infection.^12^, ^13^, ^14^

Many helminth species remain in the host for a prolonged time, and type 2 responses may be more beneficial for the survival and integrity of the host than the more inflammatory Th1/Th17 alternative. Indeed, asymptomatic infections with, for example filarial worms are associated with type 2 responses,^15^ whereas in individuals suffering from helminth infections linked to pathology and clinical symptoms, Th1 or Th17 cell responses are often found.^16^, ^17^, ^18^ In many parasitic infections, sterile immunity is not common: most individuals living in endemic areas are constantly reinfected, even after drug‐mediated clearance of parasites.^19^ As a consequence, host immune responses in endemic areas are often characterized as a “modified Th2” response that results in control (but not clearance) of parasite load, low‐level parasite transmission and minimal host pathology: an acceptable host/parasite compromise.

Tissue damage caused by helminth infections is also a powerful stimulus for type 2 responses, which in turn lead to a rapid type 2 response‐mediated healing phenotype.^20^ For example, in the lung, type 2 immune responses are important in healing damage caused by migrating *Nippostrongylus brasiliensis* larvae, while recruiting eosinophils that damage larvae, hamper their fecundity and fertility upon arrival in the gut, leading to an early expulsion.^11^ However, type 2 responses may also cause pathology due to aberrant healing, such as in the case of fibrotic granulomas formed around schistosome eggs. These granulomas cause mild to more severe pathology, linked to local fibrotic tissue, liver and splenomegaly, and an increased risk to develop cancer in the liver or the bladder, depending on the species.^21^ Thus, depending on context and infecting species, parasite products can induce epithelial cell proliferation, encourage healing, control fibrosis^22^ and cause transformation and cancerous growth.^23^

In the absence of helminth infection, type 2 responses are often perceived to be only involved in pathological allergic responses, ultimately leading to decreased lung function and airway hyperactivity in asthma and rhinitis,^24^ pruritus (itching) and damage to the skin barrier in atopic dermatitis,^25^ and itching, pain and/or swelling of the mouth, pharynx and oesophagus, diarrhoea and abdominal pain in food allergy.^26^ However, type 2 responses in the absence of helminth infections can also have beneficial roles: circulating IgE specific to venom toxins, which can cause dangerous anaphylaxis on exposure, can also be protective with release of mast cell proteases that degrade venom toxins and counteract the venom's detrimental effects.^27^ During pregnancy, the type 2 cytokine milieu in the womb protects the “non‐self” foetus from abortion (which conversely is linked to increased Th1/17 responses).^28^ Finally, perinatal type 2 responses in the lung are required for establishment of lung homoeostasis and development of anti‐inflammatory type 2 macrophages.^29^ Therefore, just as there is no such thing as “weeds” in a garden (just plants in the wrong place) perhaps there is no such thing as a “bad” immune response, just inappropriate in its context. How parasites modulate these useful and/or pathological responses, and what happens when the balance is perturbed, will be covered in the next sections.

## 3 TALES OF WORMS IN MEN

In the 1970s, the “hygiene hypothesis” was proposed as an explanation for the steep and alarming rise in the prevalence of childhood allergies and asthma among urban, Westernized societies. The hygiene hypothesis links changes in housing, sanitation and health care to increased allergic disease and proposes that this is in part due to reduced endemic infections. The prevalence of parasitic infections in particular has been drastically reduced in Westernized societies over the last century and is therefore proposed to be an important contributing factor in this hypothesis. Multiple epidemiological studies have been used to support this hypothesis by indicating that in helminth‐endemic rural areas relatively few people have allergic symptoms.^30^, ^31^ The fact that some local African languages contain no words to describe allergic symptoms could support this hypothesis, indicating that allergic diseases have never been a problem among these populations.^32^

However, an examination of the many population studies of the past 30 years either by meta‐analyses^33^, ^34^ or in some excellent systematic reviews^35^, ^36^ shows that the direction of the effect by helminth infections is far from consistent. For example, while hookworm infections were associated with a protective effect against asthma, other helminths like *Trichuris trichiura*,* Enterobius vermicularis* and *Strongyloides stercoralis* did not show any effect, and conversely *Ascaris lumbricoides* infection increased the risk of developing asthma and wheeze.^33^ Greater consensus was observed regarding protection to atopic sensitization and allergic skin reactivity, although the outcome varied with the allergen studied.^34^, ^35^, ^36^

Similarly, studies using anthelmintic treatment of helminth‐endemic populations show mixed results (for a full overview, see Wammes, 2014^35^)—some show an increased frequency of allergen skin prick test (SPT) after worm clearance,^37^ while others did not observe any differences between treatment and control groups within one or 2 year time frames.^38^ Part of the inconsistent findings and dissimilarities in conclusion in the epidemiological and interventional studies may be explained by variations in factors such as the age of the population studied, age of helminth exposure (and consequent early‐life immune imprinting) and the infectious burden, endemic parasite species and chronicity of infection, or differences in study parameters such as clinical symptoms in asthma, rhinitis or eczema or methods used to measure allergen sensitization (SPT versus allergen‐specific IgE).^35^, ^39^ Epidemiological studies applying antihelminthic treatment during pregnancy provide an interesting approach to evaluate the relationship between helminths, early immune priming and allergy: these show an increased risk of early‐life eczema in babies of treated mothers^37^, ^40^; however, a 9‐year follow‐up showed that this effect was not maintained to later life.^41^

In contrast to human studies, experiments in mice showed more consistent findings in the prevention of allergic airway inflammation by a wide range of helminth species: *N. brasiliensis*,^42^ *Heligmosomoides polygyrus*,^43^ *Litomosoides sigmodontis*,^44^ *Schistosoma mansoni*,^45^ *Trichinella spiralis* 46 and *Schistosoma japonicum*.^47^, ^48^ Interestingly, transmaternal protection against allergic airway inflammation by helminth infection in mice implied that this was dependent on the phase of the infection during pregnancy: offspring from schistosome‐infected females were protected if they had mated during the initial Th1 phase, or the chronic immune‐regulatory phases of schistosome infection, but conversely disease was exacerbated if mating occurred during the high Th2 phase of infection (coinciding with egg deposition).^49^ This may further complicate assessment of human population studies, as protection against allergy may depend on far more complicated interactions than simple presence of infection, but also prenatal stimuli and phase of infection.

## 4 HELMINTH INTERACTIONS WITH OTHER INFECTIONS AND THE MICROBIOTA

Severe respiratory syncytial virus (RSV) and/or rhinovirus (RV) infection in early life gives a sevenfold increased risk of developing asthma,^50^ while asthma exacerbations are associated with concurrent respiratory viral infection in up to 80% of cases.^51^ Furthermore, in experimental RV infections of asthmatic volunteers, IL‐33 and other type 2 cytokines were released into the airways, correlating with severity of asthma exacerbation.^52^ Parasite products can suppress IL‐33 release^53^, ^54^; thus, they could directly suppress viral proallergic responses, or, via suppression of IL‐33 release, lead to increased antiviral immune responses.^55^ Interestingly, the interactions between helminths and viruses have recently received a great deal of attention. For example, *H. polygyrus* infection leads to upregulation of type 1 interferons in the gut and lung, and suppression of respiratory syncytial virus (RSV) titre, with reduced inflammation and lung pathology in a mouse model.^56^ Likewise, *S. mansoni* infection suppresses lung pathology during pneumonia virus of mice (PVM) or influenza infection and reduced viral titre.^57^ These murine studies suggest that helminth parasites could suppress titre and/or inflammation in viral lung infections with a subsequent effect of reducing the risk of viral‐induced development or exacerbation of asthma. Though it is yet unclear whether similar effects can be found in humans, several recent virus‐helminth coinfection studies have also reported worsening of viral infection through helminth‐mediated immunosuppression; for example, *H. polygyrus* or *S. mansoni* resulted in murine γ‐herpesvirus reactivation,^58^ while *T. spiralis* infection resulted in impaired immunity to murine norovirus.^59^ Notably, in both cases, suppression of antiviral immunity was dependent on STAT‐6 and type 2 immune responses. Therefore, suppression of viral responses by helminth parasites depends on viral species and outcomes are dependent on immune mechanisms which control viral proliferation and pathology.

Helminths not only change the response to other infectious agents within the host but can also affect the balance of commensal organisms with which they share an environment. The host genome and the total diversity of the microbiota (the “microbiome”) are important in mediating or reflecting health and disease in the intestine, and in other barrier sites such as the lung and skin. Changes in the gut and lung microbiomes are seen in allergic diseases such as asthma,^60^ reflecting the immune axis between these mucosal sites or transfer of bacterial populations through processes such as inhalation of airborne bacteria, bacterial migration along mucosal surfaces and microaspiration of gastric contents.^61^

Although many studies of the microbiome focus on faecal contents, it is important to note that intestinal helminths infect specific niches within the intestine: that is *Trichuris* spp in the large intestines, and human hookworms, *H. polygyrus* and *N. brasilienesis* in the small intestines. In humans living in helminth‐endemic areas and during experimental helminth infection, helminth infection is associated with increased diversity and abundance of the microbiome.^62^, ^63^, ^64^, ^65^ Although it has not been demonstrated whether these differences are related to differences in lifestyle and hygiene or causally linked to current helminth infection, the fact that changes in the microbiota are partially abrogated on anthelmintic treatment supports the latter hypothesis.^63^ In several mouse models, intestinal helminth infections induced a decreased prevalence of commensals associated with inflammation^66^, ^67^ and increase in commensals associated with immune regulation.^63^, ^68^ This is proposed to be an active process, mediated by secreted antimicrobials from the parasite (such as host defence peptides),^69^ or mediated by (type 2) immune responses directed against or modulated by the parasite.^63^, ^70^ Consequently, changes in the microbiota (due to, eg changes in diet) and microbial metabolite levels (such as short‐chain fatty acids) mediate changes in allergic responsiveness.^71^ Interestingly, a recent study also found altered fatty acid production by the microbiota of *H*. *polygyrus*‐infected mice and their additional role in protection against allergic airway inflammation.^72^

## 5 REWORMING THE WEST

The growing support for the idea that helminth infections suppress inflammatory responses led to the proposal of using helminth infections as therapeutic agents in these diseases. In the first clinical trials of “helminth therapy,” patients with inflammatory bowel disease (Crohn's disease or ulcerative colitis) were treated with eggs (ova) from the porcine intestinal parasite *Trichuris suis* (TSO), leading to significant reduction in symptom scores in a series of small trials.^73^, ^74^, ^75^ Likewise, an observational study in Argentina showing that multiple sclerosis (MS) patients went into remission after infection with environmentally acquired helminths,^76^, ^77^ and when treated to clear their helminth infections, their autoimmune disease was again reactivated.^78^ Finally, a series of studies have used human hookworm (*Necator americanus*) in patients with coeliac disease: although initial studies showed no clinically significant difference in responses to gluten challenge,^79^ inflammatory immune responses in the gut were reduced and skewed towards a Th2 response.^80^, ^81^ In a follow‐up open‐label study using escalating doses of gluten after hookworm infection, no deterioration in clinical pathology was seen in hookworm‐infected subjects^82^ giving grounds for further studies.

These initial studies formed the basis of clinical trials treating patients with IBD or multiple sclerosis in the United States and Europe with TSO. However, to date, these trials have shown disappointing response rates and no significant reduction compared with placebo controls.^83^, ^84^, ^85^

Of most relevance to this review, studies using hookworm infection to treat asthma or TSO to treat allergic rhinitis patients have also been undertaken. However, no change in clinical measurements was seen in either study,^86^ and although type 2 specific responses were detected against the hookworm, no regulatory immune responses were found.^87^

**Figure 1:**
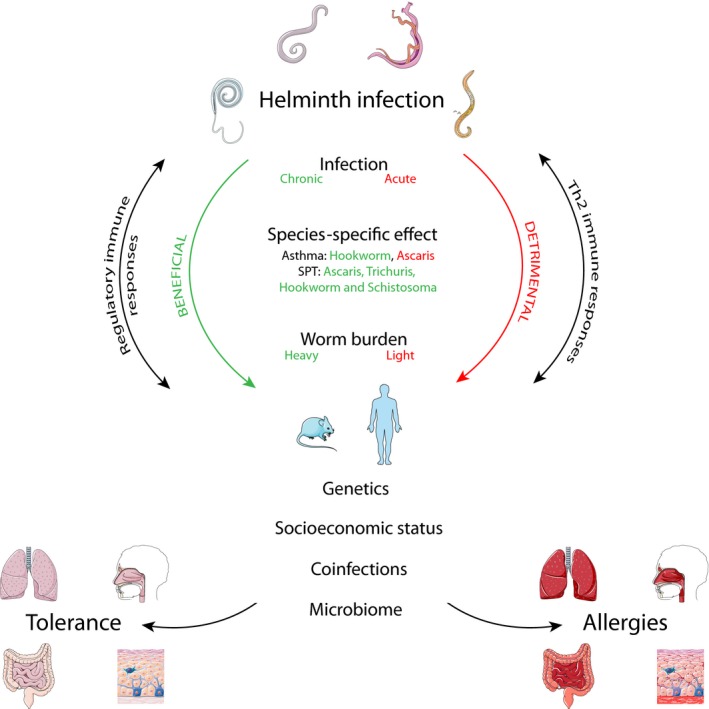
Helminth infections are associated with both promoting and reducing allergic symptoms. Helminths interact strongly with the host immune system, but the type of response is heavily influenced by the chronicity of infection, the species involved and/or worm burden, ultimately tipping the balance towards more detrimental and type 2 response or beneficial and regulatory responses. Subsequently, this balance is further influenced by cofactors such as host genetics, socioeconomic status, coinfections and the composition and diversity of the microbiome leading to the development of clinical symptoms and allergies or tolerance in the host. Image is adapted from Servier Medical ART

Thereby, the promise of helminth therapy has so far not translated to a practicable treatment for human disease. Reasons for this may well include difference between prevention and cure (ie parasitic infection may need to precede allergic sensitization, and effect could be in utero^49^), the difference between infection and administration of parasite products (most of the therapeutic effects in mouse models were based on the application of helminth products rather than a full infection), the single parasite infective dose given (which is generally determined by that which causes no notable side effects, but may therefore be too low and too little for functional suppression of pathology) or disease endotypes which are responsive or refractory to these treatments, precluding statistical significance of effects when the disease population is taken as a whole.^88^ Critically, however, the mechanism of action of helminth immunomodulation is not well understood, and whether this is shared between all helminth infections, or more likely unique to each parasitic species, is presently unknown (Figure 1).

**Figure 2:**
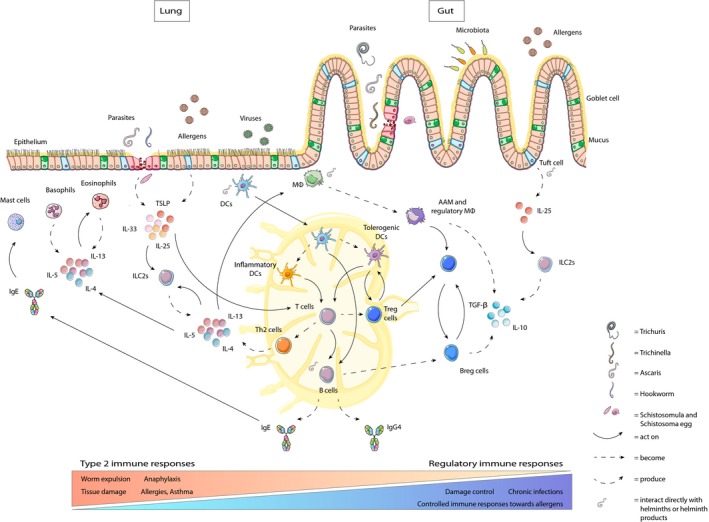
Immune responses during helminth infections. Depending on their life cycle, various helminth species will pass or reside in (the proximity of) the lung and the gut. Consequently, damage will occur, leading to the release of alarmin cytokines IL‐33, IL‐25 and thymic stromal lymphopoietin (TSLP) by epithelial cells and tuft cells (gut). These cytokines will act on innate lymphoid cells (ILC)2 and on dendritic cells (DCs), which will migrate to the draining lymph node and skew naïve T cells towards polarized Th2 cells, producing the cytokines IL‐4, IL‐5 and IL‐13, in a similar fashion as ILC2s. These cytokines are central to the type 2 immune response and drive the isotype switch to IgE immunoglobulins, act on eosinophils, mast cells and drive the development of alternatively activated macrophages. All these elements are instrumental in worm expulsion but can also promote tissue damage, anaphylaxis and allergic responses towards bystander antigens. These responses are balanced by various cells from the regulatory network: for example regulatory T and B cells, regulatory macrophages and tolerogenic DCs. These regulatory cells can act on other cell types directly or through the production of anti‐inflammatory cytokines IL‐10 and TGF‐β, as well as by the induction of anti‐inflammatory IgG4, leading to immune tolerance and damage control, but at the same time prevent worm expulsion, promoting chronic helminth infections. Image is adapted from Servier Medical ART

In the following sections, we will focus on mechanisms by which type 2 immune responses are suppressed or induced in helminth infections, and how this could affect allergic responses (Figure 2 for a schematic overview). A deeper understanding of the interaction between helminths and their host will help to translate these mechanisms into a better therapeutic approach.

## 6 HELMINTHS AND MODULATION OF ALLERGIC DISEASE: THE ROLE OF IMMUNOGLOBULINS, REGULATORY CELLS AND CYTOKINE INHIBITORS

### 6.1 Immunoglobulins

Allergen‐specific IgE is the defining characteristic of atopy, and in high‐income countries, allergen‐specific IgE strongly correlates with functional allergy measured by SPT reactivity. However, in helminth‐endemic areas (especially rural areas of low socioeconomic status), this relationship often breaks down.^89^, ^90^ Furthermore, multiple epidemiological studies have shown a positive association between antihelminth IgE (ascariasis, schistosomiasis, filariasis) and wheeze and/or atopy.^91^, ^92^, ^93^, ^94^, ^95^ One of the reasons for discrepancies between IgE reactivity (against allergen or helminth) and allergy in high‐income versus helminth‐endemic areas might be due to cross‐reactivity of antihelminth IgE to certain allergens. For example, IgE against tropomyosin from *Onchocerca volvulus* cross‐reacts with the tropomyosin of house dust mite Der p 10, boosting allergic responses to HDM.^96^, ^97^ While IgE against carbohydrates on Schistosoma egg glycoproteins can cross‐react with cross‐reactive carbohydrate determinants (CCDs) on peanut antigens, due to the low affinity of this IgE, cross‐linking and degranulation of carbohydrate‐specific IgE‐coated mast cells do not occur. Therefore, these cross‐reactive responses have the potential to block clinical responses to allergens like peanut.^98^ Further studies are needed to clarify whether high levels of circulating cross‐reactive protein or carbohydrate‐specific IgE are instrumental in inducing or preventing allergic (skin) reactivity.^89^

The immunoglobulin isotype IgG4 is often associated with a tolerized allergic response following allergen‐specific immunotherapy, and its production is also increased in many helminth‐infected individuals.^99^, ^100^ Although there is a growing awareness of potentially harmful effects of IgG4 in several IgG4‐related systemic diseases,^101^ it is unclear how this pathogenic role of IgG4 compares to active tolerance induction to allergens or during helminth infection. Despite these recent reports on IgG4‐related diseases, IgG4 antibodies are considered the least inflammatory of all isotypes—they do not activate complement, and unlike IgE they do not cause degranulation of mast cells. Due to the unique ability of IgG4 to swap antigen‐binding arms, it is regarded as functionally monovalent and thus will not cause immune complex formation.^100^ Thus, in this context, its main function appears to be a blocking one, and possibly instrumental in preventing IgE‐mediated inflammation. High levels of anti‐*Ascaris* IgG4 have been negatively associated with allergen SPT positivity,^102^ while in a *S. mansoni*‐endemic area—although higher levels of both IgE and IgG4 were found in infected individuals—a higher ratio of IgE to IgG4 predicted clinical allergic symptoms,^103^ just as in allergen‐specific immunotherapy.^104^ As many helminth products are homologous to common allergens, IgG4 responses raised against helminth products may also bind and block IgE epitopes on allergens, reducing responses to allergens and directly reducing SPT responses.^105^, ^106^, ^107^ Mechanistic research into the role of IgE and/or IgG4 is hampered by the lack of good experimental animal models, as IgG4 does not exist in mice.

### 6.2 Regulatory cells

Both regulatory T cells (Tregs) and regulatory B cells (Bregs) are important in the control of type 2 immune responses and allergic airway inflammation in mouse models.^108^ In individuals tolerized to allergens through high‐dose environmental exposure or allergen‐specific immunotherapy, levels of Tregs and Bregs are increased and required for maintenance of tolerance.^108^ Both Tregs and Bregs can produce the immunosuppressive cytokines IL‐10 and TGF‐β, which can suppress damaging inflammation.^109^

IL‐10 and TGF‐β are also instrumental in immunosuppressive effects in a number of different helminths, including *Onchocerca*,* Ascaris*,* Trichuris* or *Toxocara* spp,^110^, ^111^, ^112^ and appear to be important in suppression of allergic responses. Indeed, IL‐10 was linked to a lower risk of allergic skin reactivity in schistosome‐infected Gabonese schoolchildren.^113^ Elevated numbers of circulating FOXP3+ CD25+ Treg cells have been demonstrated in *Schistosoma haematobium* 114 and filaria‐infected people,^115^ while anthelmintic treatment of *S. haematobium* or geohelminth‐infected individuals leads to a normalization of circulating FOXP3 Treg or PD‐1 and CTLA‐4‐expressing CD4^+^ T cells^116^ and/or subsequent increased in vitro cytokine responses to both helminth and bystander antigens.^114^ Similarly, increased levels of Breg cells have been detected in helminth‐infected MS patients^77^ and in *S. haematobium*‐infected Gabonese people.^45^, ^117^

Also in mouse models of allergic airway inflammation (AAI), both helminth‐induced Treg and Breg cells are instrumental in preventing disease symptoms. For example, Tregs from *H. polygyrus* or *T. spiralis*‐infected mice transferred protection against airway pathology in models of experimental airway allergy.^43^, ^46^, ^118^ *Heligmosomoides polygyrus* excretory/secretory products (HES) can induce Tregs in vitro, and transfer of HES‐induced Tregs can replicate the suppressive capacity of the parasitic infection.^119^ Recently, a TGF‐β mimic (Hp‐TGM) was identified in HES, a protein which alone can induce Tregs in vitro.^120^, ^121^

Likewise, mesenteric lymph node CD23^hi^ B cells from *H. polygyrus*‐infected mice suppress allergic airway inflammation in an IL‐10‐independent manner,^122^ while splenic marginal zone CD1d^hi^ B cells from *S. mansoni*‐infected mice induced protection in an IL‐10 and Treg cell‐dependent manner upon adoptive transfer.^45^, ^123^ Analysis of splenic CD1d^hi^ B cells from schistosome‐infected mice showed increased *Tlr7* expression, and TLR‐7 ligation increased the IL‐10 production in splenic CD1d^hi^ B cells from naïve animals.^124^ Adoptive transfer of TLR‐7 stimulated splenic CD1d^hi^ B cells reduced allergic airway inflammation through the recruitment of regulatory T cells. Further mechanistic insight was recently supplied by the finding that the *S. mansoni*‐derived molecule IPSE/alpha‐1 could drive Breg differentiation in vitro.^125^ In addition, and separately to “conventional” IL‐10‐producing Bregs, *S. mansoni*‐infected mouse lungs also contain a nonclassical regulatory B‐cell population that could also inhibit AAI by reducing allergen‐specific Th2 responses, in an IL‐10 and Treg‐independent manner.^126^

These studies point towards an important role for helminth‐induced Tregs and Bregs in the suppression of allergen‐specific immune responses. However, part of these processes may also be accelerated by exhausted and hyporesponsive T‐ and B‐cell responses.^127^, ^128^, ^129^, ^130^ For example, in murine *L. sigmodontis* infection, Th2 cells upregulate GITR, CTLA‐4 and PD‐1 and become hyporesponsive to stimuli,^131^, ^132^, ^133^, ^134^ while in chronic murine schistosome infection, hyporesponsive Th2 cells were linked to the anergy marker GRAIL.^135^ Thus, in chronic Th2 dominated models, such as helminth infection, Th2 cells become hyporesponsive and anergic. This is to the benefit of both the parasite (allowing survival) and, often, the host (preventing inflammatory damage). Likewise, in allergen immunotherapy, through either anergy or deletion, Th2 cells decrease in number, reducing IL‐4, IL‐5 and IL‐13 production.^136^ Thus, hyporesponsive Th2 cells may share similar features in chronic helminth infection and tolerized allergic responses, however, whether this is an active process, and whether helminth infection causes hyporesponsiveness in bystander allergic responses in vivo is presently unclear.

### 6.3 Myeloid cells

Dendritic cells (DC) are the critical link between innate and adaptive immunity and decide on the development of effector versus regulatory T‐cell development based on their ontogeny, tissue location and/or environmental signals present. Different myeloid DC subsets—conventional type 1 (cDC1) and type 2 dendritic cells (cDC2)—can be distinguished on the basis of several surface expression markers recently identified in an unbiased approach across tissues and species^137^, ^138^. While cDC1 can produce high levels of IL‐12p70 and prime cytotoxic CD8 T‐cell and antitumour responses, cDC2 can boost both Th17 or Th2 cells depending on the environment and show superior allergen uptake compared to cDC1.^139^, ^140^, ^141^, ^142^, ^143^, ^144^ Interestingly, cDC1 can also have a tolerogenic function in allergy models: they induce Treg cells via retinoic acid and peroxisome proliferator‐activated receptor gamma (PPARγ) and limit inflammation in a HDM and ovalbumin model of allergic airway inflammation^145^ and during schistosome infections.^146^ In patients with asthma, increased numbers of DCs are found in the blood, induced sputum and bronchoalveolar lavage upon allergen challenge, but only cDC2 migrated into the bronchial tissue.^147^ The DCs express more OX‐40L, a molecule involved in Th2 polarization^148^ and more FcεRI.^149^ In the absence of DCs, type 2 responses in allergy models are profoundly abrogated,^150^ but other myeloid cell populations may also be important to support Th2 cell development in either allergy models or helminth infection, like monocyte‐derived dendritic cells.^140^, ^141^

Macrophages differentiate into alternatively activated or M2 macrophages in response to IL‐4 and IL‐13. They can be distinguished from classically activated or M1 macrophages by the upregulation of markers such as RELM‐α, Ym1 and arginase and are associated with wound healing and parasite killing.^11^, ^151^ M2 macrophages are also anti‐inflammatory, producing IL‐10 and TGFβ^152^ and arginase, which restrict T‐cell function through amino acid starvation^153^ and suppress liver fibrosis in *S. mansoni* infection.^154^ Furthermore, retinoid acid production by M2 macrophages during *S. mansoni* infections promotes the development of Treg cells at the sites of inflammation.^155^ In allergic asthma, macrophages are considered to play a key role in inflammatory responses associated with lung injury, fibrosis and goblet cell hyperplasia^156^ and stimulating smooth muscle cell contraction and extracellular cell matrix degradation, contributing to airway remodelling. Increased numbers of mannose receptor (MR)+ macrophages (a surface marker for M2 macrophages) are found in the bronchial tissue of allergic asthmatic patients.^157^

Immature or tolerogenic dendritic cells—induced by immunosuppressive drugs or molecules, like vitamin D3^158^—are potent drivers of regulatory T cells and immune tolerance. The ES products of *Ancylostoma caninum* can suppress immunopathology in mouse models of colitis.^159^ Recently, AIP‐1 (from the human hookworm *N. americanus*) and AIP‐2 (from *A. caninum*) were shown to be suppressive in mouse models of colitis^160^ and asthma^161^ by a mechanism dependent on Treg expansion. Although the full mechanism of action of these molecules has yet to be elucidated, it appears that they achieve Treg expansion through modulation of dendritic cell responses.^161^

Likewise, regulatory macrophage populations can express IL‐10 that is instrumental in Treg development and/or suppression of local immune responses. Both regulatory DC and macrophages are heavily exploited in immune evasive strategies by various pathogens.^162^ This suppression also occurs in vivo, with changes in DC maturation status and phenotype and Treg‐inducing function during experimental murine helminth infection,^163^, ^164^, ^165^ with helminth product‐induced macrophage IL‐10 expression^166^, ^167^ and in human helminth‐endemic populations.^168^, ^169^ In addition, adoptive transfer of tolerogenic DCs from *S. japonicum*‐infected or *H. polygyrus*‐infected mice reduced ovalbumin‐induced AAI or colitis in uninfected recipients via IL‐10.^165^, ^170^ Thus, both macrophages and DCs are capable of differentiating into inflammatory or suppressive phenotypes, both pathways being prone to helminth immunomodulation.

Both macrophages and DCs are modulated by parasite products through pattern recognition receptors (PRR), such as the TLR family and the C‐type lectin receptors. Helminths secrete many molecules, a significant majority of which are decorated with glycans that play a role in parasite‐host interactions.^171^ The failure of live hookworm or TSO clinical trials has fuelled the interest in the immunomodulatory properties of helminth molecules and their glycans as potential therapeutic agents. Examples of immunomodulatory glycans or glycoproteins in the modulation of dendritic cell function and/or disease models are described for glycans from *Trichuris* eggs in TSO, the *S. mansoni* egg glycoprotein, omega‐1—a T2 RNase—and the glycoprotein ES‐62 secreted by *Acanthocheilonema viteae*.^172^, ^173^, ^174^, ^175^

Other molecules that act on dendritic cell/macrophage function and dampen experimental allergic airway inflammation are identified from *Clonorchis sinensis, A. caninum, A. viteae*,* Brugia malayi* and *Anisakis simplex*.^161^, ^166^, ^167^, ^176^, ^177^, ^178^ Further studies are needed to clarify whether these molecules can also be used in allergic and asthmatic patients in a therapeutic setting without the disadvantages of the infection itself.

### 6.4 Innate lymphoid and epithelial cells

The importance of early, innate, epithelial cell‐derived cytokines in type 2 response initiation has only recently begun to be understood. The epithelial cell cytokines IL‐25, IL‐33 and TSLP activate ILC2s at barrier sites, which secrete large amounts of IL‐5, IL‐13 and IL‐9. Recently, it was shown that ILC2s can also be activated to produce IL‐10, providing a immunoregulatory pathway (similar to that seen in T cells) which could be amenable to parasite immunomodulation.^9^

Proximal epithelial cell responses therefore represent an ideal target for intervention, as blocking these cytokines could blunt downstream ILC, dendritic cell and T‐cell responses. However, as these pathways have only recently been characterized, research into their suppression remains in its infancy. One exception is the IL‐33 pathway in *H. polygyrus* infection, in which multiple parasite immunomodulatory factors have been identified. *Heligmosomoides polygyrus* excretory/secretory products (HES) replicate the suppressive effect of parasitic infection in suppression of airway allergic inflammation,^179^ via abrogation of the earliest ILC2 responses to an allergen preparation from *Alternaria alternata,* 53 a clinically relevant stimulus. *Alternaria* allergen administration is a uniquely potent stimulus for IL‐33 release,^180^ and HES administration blocks the IL‐33 pathway through induction of IL‐1β (counter‐regulating IL‐25 and IL‐33 expression),^181^ miRNA‐containing HES extracellular vesicles which reduce IL‐33 receptor expression, and HpARI, a protein in HES which directly binds IL‐33, abrogating its release.^54^

Recently, it was shown that cholinergic neurons activate^182^, ^183^, ^184^ and adrenergic neurons inhibit^185^ ILC2 responses, while type 2 cytokines in turn activate neurons,^186^ forming a new field of neuroimmune interactions for future studies, and potential helminth modulation.

## 7 CONCLUDING REMARKS

Helminth parasites have coevolved with their mammalian hosts for millions of years, and in doing so have developed an intimate relationship. On the one hand, this relationship can be seen as antagonistic, with the parasite attempting to subvert immune responses to its own ends, while the host immune system attempts to damage or kill the parasite. Alternatively, it can be seen as a mutualistic interaction, with parasite immunomodulation expected by the host immune system, and in fact required for healthy immune development, and avoidance of immune‐mediated disease. It is likely that both forms of interaction occur, depending on context, parasite and environment. “Gene‐environment” interactions in allergy and helminth infections are further complicated by “infection‐environment” interactions with diet, living conditions depending on the socioeconomic status and/or rural versus urbanization sanitation, microbiota and coinfections, all of which have roles in the response to, and tolerance of, allergenic stimuli. By learning how, at the molecular level, these interactions occur; we may be able to replicate and tailor beneficial helminth‐mediated effects for use in allergic disease, in the absence of the deleterious effects that come with parasitic infection.
